# Genome-Wide Identification and Characterization of UTR-Introns of *Citrus sinensis*

**DOI:** 10.3390/ijms21093088

**Published:** 2020-04-27

**Authors:** Xiaobao Shi, Junwei Wu, Raphael Anue Mensah, Na Tian, Jiapeng Liu, Fan Liu, Jialan Chen, Jingru Che, Ye Guo, Binghua Wu, Guangyan Zhong, Chunzhen Cheng

**Affiliations:** 1 College of Horticulture, Fujian Agriculture and Forestry University, Fuzhou 350002, China; 2 Institute of Fruit Tree Research, Guangdong Academy of Agricultural Sciences, Guangzhou 510640, China

**Keywords:** UTR intron, nucleotide distribution, *Cis*- elements, gene expression

## Abstract

Introns exist not only in coding sequences (CDSs) but also in untranslated regions (UTRs) of a gene. Recent studies in animals and model plants such as *Arabidopsis* have revealed that the UTR-introns (UIs) are widely presented in most genomes and involved in regulation of gene expression or RNA stability. In the present study, we identified introns at both 5′UTRs (5UIs) and 3′UTRs (3UIs) of sweet orange genes, investigated their size and nucleotide distribution characteristics, and explored the distribution of *cis*-elements in the UI sequences. Functional category of genes with predicted UIs were further analyzed using GO, KEGG, and PageMan enrichment. In addition, the organ-dependent splicing and abundance of selected UI-containing genes in root, leaf, and stem were experimentally determined. Totally, we identified 825 UI- and 570 3UI-containing transcripts, corresponding to 617 and 469 genes, respectively. Among them, 74 genes contain both 5UI and 3UI. Nucleotide distribution analysis showed that 5UI distribution is biased at both ends of 5′UTR whiles 3UI distribution is biased close to the start site of 3′UTR. *Cis-* elements analysis revealed that 5UI and 3UI sequences were rich of promoter-enhancing related elements, indicating that they might function in regulating the expression through them. Function enrichment analysis revealed that genes containing 5UI are significantly enriched in the RNA transport pathway. While, genes containing 3UI are significantly enriched in splicesome. Notably, many *pentatricopeptide repeat-containing protein genes* and the *disease resistance* *genes* were identified to be 3UI-containing. RT-PCR result confirmed the existence of UIs in the eight selected gene transcripts whereas alternative splicing events were found in some of them. Meanwhile, qRT-PCR result showed that UIs were differentially expressed among organs, and significant correlation was found between some genes and their UIs, for example: The expression of *VPS28* and its 3UI was significantly negative correlated. This is the first report about the UIs in sweet orange from genome-wide level, which could provide evidence for further understanding of the role of UIs in gene expression regulation.

## 1. Introduction

The existence of intron in genes was initially discovered in late 1970 [1]. Gradually, it has come to be identified as a common event in all eukaryotic genomes. In the past, it was recognized as an important gene structure that was eliminated from transcripts by a complex molecular mechanism called a spliceosome [2,3,4]. Recently, however, the intron retention (or alternative splicing, AS) in transcripts has also been widely identified, suggesting that they are not only components in gDNA but may also play roles in gene expression regulation or even functioning.

Introns are important for the gene expression process and are thought to go through five phases before the formation of mature mRNA: Genomic intron, transcribed intron, spliced intron, excised intron and exon-junction complex (EJC)-harboring transcript [4]. The presence and removal of introns in gene transcripts affect almost every step of gene expression, including transcription and polyadenylation, mRNA export, localization, translation and attenuation [5,6]. Leon et al. [7] reported that the maize *Adhl* intron 1 enhanced gene expression in the legume species for about 10-fold. The *Sh1* intron 1 enhances chimeric gene expression in rice and maize protoplasts approximately 100-fold [8]. These have revealed that the introns in the transcripts showed positive effect on gene expression, i.e., intron-mediated enhancement (IME) [9,10,11].

In eukaryotes, mature mRNAs have a tripartite structure consisting of a 5′-untranslated region (5′UTR), a coding region (CDS) and a 3′-untranslated region (3′UTR). It has been widely proven that UTRs play crucial roles in the control of mRNA translation efficiency, stability and subcellular localization [12]. There are also introns in the 5′ and 3′ UTRs of many protein-coding genes. The 5′UTR intron (5UI) is usually longer than the intron within the CDS and may affect gene expression, mRNA stability or mRNA export [13]. Reports have shown that, like CDS introns close to the 5′ end of a gene, introns located in the 5′UTR also often exert positive effects on gene expression by affecting the basal promoter activity [14,15,16,17,18]. In the study of Norris et al. [19], the 5UIs of *Arabidopsis thaliana* polyubiquitin genes (*UBQ3*, *UBQ10,* and *UBQ11*) were identified to be quantitative determinants of chimeric gene expression. Grant et al. [16] reported that the 5UI might contribute to the high activity of the soybean polyubiquitin promoter. Sivamani and Qu [20] reported that the rice polyubiquitin gene (*rubi3*) promoter containing the 5UI conferred approximately 20-fold higher *GUS* gene expression than the intron-less promotor in bombarded rice suspension cells. Chaubet-Gigot et al. [21] demonstrated that the 5UI of Arabidopsis *replacement histone H3* genes together with their endogenous promoters could produce higher expression of the *H3* genes. Kamo et al. [15] also revealed that transgenic *Gladiolus* (monocot) and *Arabidopsis* (dicotyledon) plants overexpressing the *Gladiolus polyubiquitin* promoter (*GUBQ1*) containing 5UI showed higher *GUS* expression level compared with the transgenic plants overexpressing *GUBQ1* promoter without it.

Unlike 5UI, 3UI was thought to usually downregulate gene expression levels [22,23] through a nonsense-mediated decay (NMD) pathway [24,25]. The NMD inhibition affects a much higher proportion of 3UI containing gene transcripts than non-3UI containing gene transcripts, and the most significantly enriched NMD-affected gene transcripts are those that encode RNA-binding proteins [23,26,27]. The transcripts containing 3UIs were previously thought to be nonfunctional because of their rapid degradation through NMD. Recently, however, some 3UI containing transcripts were also proved to be functional [23].

The genome-wide identification of UIs can provide information for the understanding of gene expression regulation and alternative splicing network in the species from the whole genome level. Cenik et al. [28] studied the UI distribution in human genome and found that the highly expressive genes often had a short 5UI, indicating the 5UI in the human genome enhanced the expression of certain genes in a length-dependent manner. Hong et al. [29] analyzed the intron size, abundance, and distribution in *A. thaliana*, *Drosophila melanogaster*, human, and mouse UTRs, found that 5UIs were approximately twice as large as introns in CDSs and 3UIs, and there was a sharp drop in intron size at the 5′UTR-CDS boundary. Chung et al. [30] analyzed the UI distribution in *A. thaliana* genes, found that 5UIs from Arabidopsis were more proximal to the UTR end or closer to the transcriptional start site. Moreover, they also found that the 5UI in *EF1a-A3* gene could enhance its expression in a size dependent manner.

Sweet orange (*Citrus sinensis*) is an important fruit with high nutrition and economical values. It accounts for approximately 60% of citrus production for both fresh fruit and processed juice consumption (FAO, http://faostat.fao.org/default.aspx). Its genome has been published and the UTR information was well addressed [31], which could be used for the genome-wide identification of UIs. Based on the *C*. *sinensis* genome, we identified and characterized the distribution of introns, including 5UI, 3UI, and introns in CDSs. The UI existence conditions in several genes were determined using RT-PCR and their expression pattern in different organs were investigated using qRT-PCR. To our knowledge, this is the first report of horticultural plant UIs from genome-wide level.

## 2. Results and Discussion

### 2.1. UTR Introns in the Citrus sinensis Genome

Totally, 29,655 protein-coding genes were identified from the sweet orange genome, of which 15,823 (53.36%) contained both 5′UTR and 3′UTR, 1093 (3.69%) only contained 5′UTR, 1585 (5.34%) only contained 3′UTR, and 11,154 (37.61%) contained neither. In *Arabidopsis*, more genes were found to lack 5′ UTR annotation rather than 3′ annotation [30], which is similar to the result found in our present study. To show the intron density in UTRs and CDSs, we normalized the intron density to the average number of introns per nucleotide of each gene transcript sequence. The intron density followed the order: CDS > 5′UTR > 3′UTR, which was 1.42 × 10^−4^, 2.98 × 10^−3^ and 6.36 × 10^−5^ respectively (Table 1). The intron density in 5′UTRs is ~2.2 times the intron density in 3′UTRs and is only ~4.8% of the intron density in CDSs. The intron density in the UTRs and CDSs of Arabidopsis gene transcripts also follow the same order, but with much higher density [30]. Notably, the intron density in the Arabidopsis 5′UTRs (~1.6 × 10^−3^, about 60% of CDSs) was > 10 times higher than that of sweet orange, indicating that the 5UI regulation of gene expression in Arabidopsis is more frequent. The high 5′UTR intron density might be due to the fact that the Arabidopsis genome is better sequenced and annotated with less unknown sequences (Ns). In both sweet orange and Arabidopsis, the 3′UTR intron density is the lowest (the 3UI density is only), suggesting that plants may utilize a similar NMD pathway like mammals [30,32,33].

A total of 965 5UIs and 745 3UIs respectively were identified from 825 5′UTR- and 570 3′UTR-containing gene transcripts, corresponding to 617 and 469 genes (Supplemental Materials Table S1), respectively. Among these UI-containing gene transcripts (UI-Ts), 77 (74 genes) were found to contain both 5UI and 3UI. Around 86.43% of the 5UI-Ts and 78.60% of the 3UI-Ts only contain one 5UI or 3UI (Table 2). The proportion of UI-Ts with two or more UIs dropped greatly, which is consistent with previous reports in other organisms [28,29,34]. Only 10.91% of the 5UI-Ts contain two 5UIs, and 15.79% of the 3UI-Ts contain two 3UIs. The percentages of UI-Ts containing more than three 5UIs or 3UIs were both less than 4%. Notably, we also identified some UI-Ts were of several UIs, for example, a *A20/AN1-like zinc finger family protein* gene (Cs7g06380.1) and an unknown gene (orange1.1t06039.1) had five 5UIs; a *zinc finger protein* gene (Cs2g17870.1) and another unknown gene (Cs5g25765.1) had four 5UIs; two *NB-ARC domain-containing disease resistance protein* genes (Cs1g15550.1, Cs1g17000.1) had six 3UIs, four *pentatricopeptide repeat* (*PPR*)*-containing protein* genes (Cs2g11780.1, orange1.1t01460.1, orange1.1t03486.1,) and a *NB-ARC domain-containing disease resistance protein* gene (Cs1g16990.1) had five 3UIs, eight unknown genes (Cs2g08495.1, Cs3g12715.1, orange1.1t05956.1, orange1.1t03536.1), one *S-phase kinase-associated protein 1* (Cs3g10260.1), one *pentatricopeptide repeat (PPR)-containing protein* gene (Cs5g26550.1), one *NB-ARC domain-containing disease resistance protein* gene (Cs5g28645.2) and one *tetratricopeptide repeat (TPR)-like superfamily protein* gene (Cs7g15390.1) had four 3UIs. Interestingly, by searching the expression data of sweet orange genes in the genome database, we found that the UI-Ts containing multiple 3UIs usually tend to record low-expression levels (average FPKM < 4).

**Figure 1:**
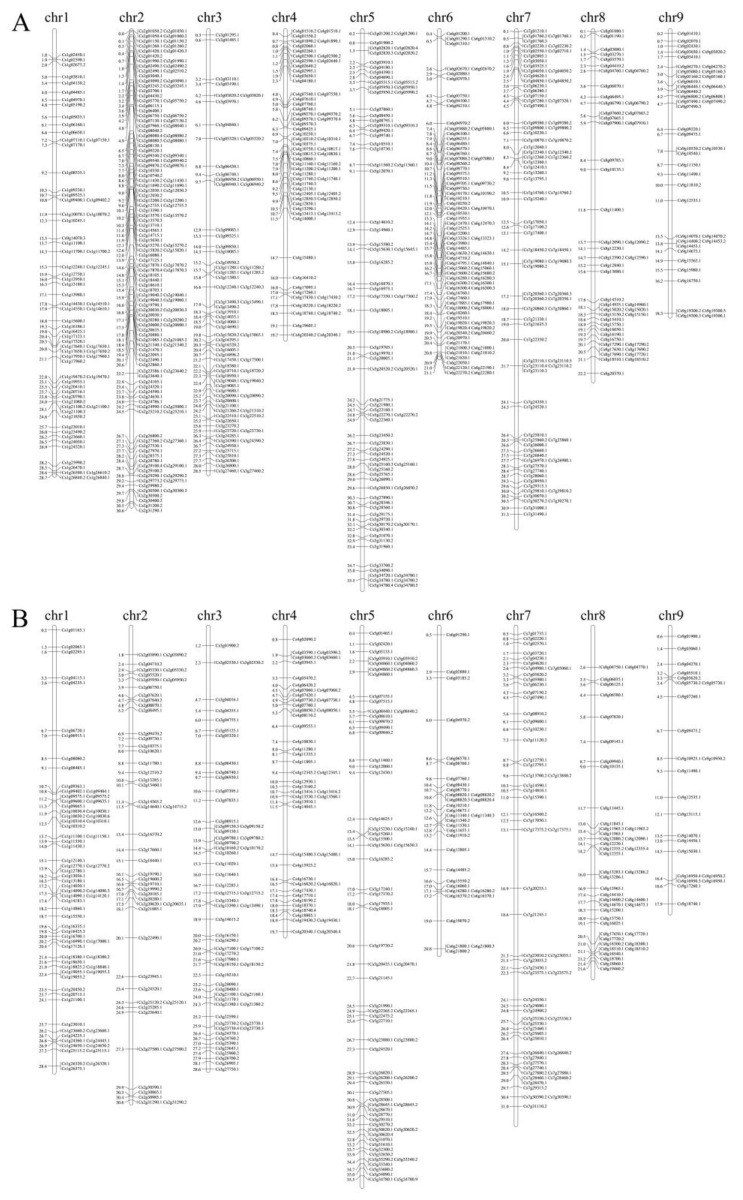
Chromosome localization results of gene transcripts containing 5′UTR intron (5UI) (**A**) and 3′UTR intron (3UI) (**B**) in *Citrus sinensis*. Chr: chromosome, cM: centiMorgan.

We further mapped the UI-Ts to the sweet orange chromosomes (Figure 1). Results showed that the UI-Ts were unevenly distributed in all the sweet orange chromosomes. Chromosome 2 showed the highest 5UI-T distribution (16.79%) and the highest density (5.26 × 10^−6^), while chromosome 1 showed the highest 3UI-T distribution (13.83%) and the highest density (3.58 × 10^−6^) (Table 3). Chromosome 9 contained the least 5UI-Ts and 3UI-Ts, respectively accounting for 4.96% and 3.62% (Table 3). The 5UI-Ts densities in chromosome 2, 3, 4, 5, 6, 7, and 9 were all higher than the 3UI-Ts densities. Notably, the 5UI-T density is about 2.75 times higher than the 3UI-T density in chromosome 2, and 2.57 times higher in chromosome Moreover, the UI-Ts distribution in the same chromosome is also uneven. For example, significant more 5UI-Ts were found in the 3′ ends of chromosome 3, 5, and 6 and in the 5′ end of chromosome 4, and significantly more 3UI-Ts in the 3′ ends of chromosome 1, 2, 5, and 8.

We further calculated the UI distribution in the sweet orange chromosomes. Like the UI-T distribution result, the highest 5UI density was found in Chromosome 2 and the highest 3UI density was found in Chromosome 1, and the lowest 3UI density (2.38 × 10^−6^) was found in chromosome 9 (Table 3). The 5UI density in chromosome 2 and chromosome 6 were both ~2 times of 3UI density. However, unlike the 5UI-T distribution condition, the lowest 5UI density was found in chromosome 8, which might be due to the chromosome length differences.

**Figure 2:**
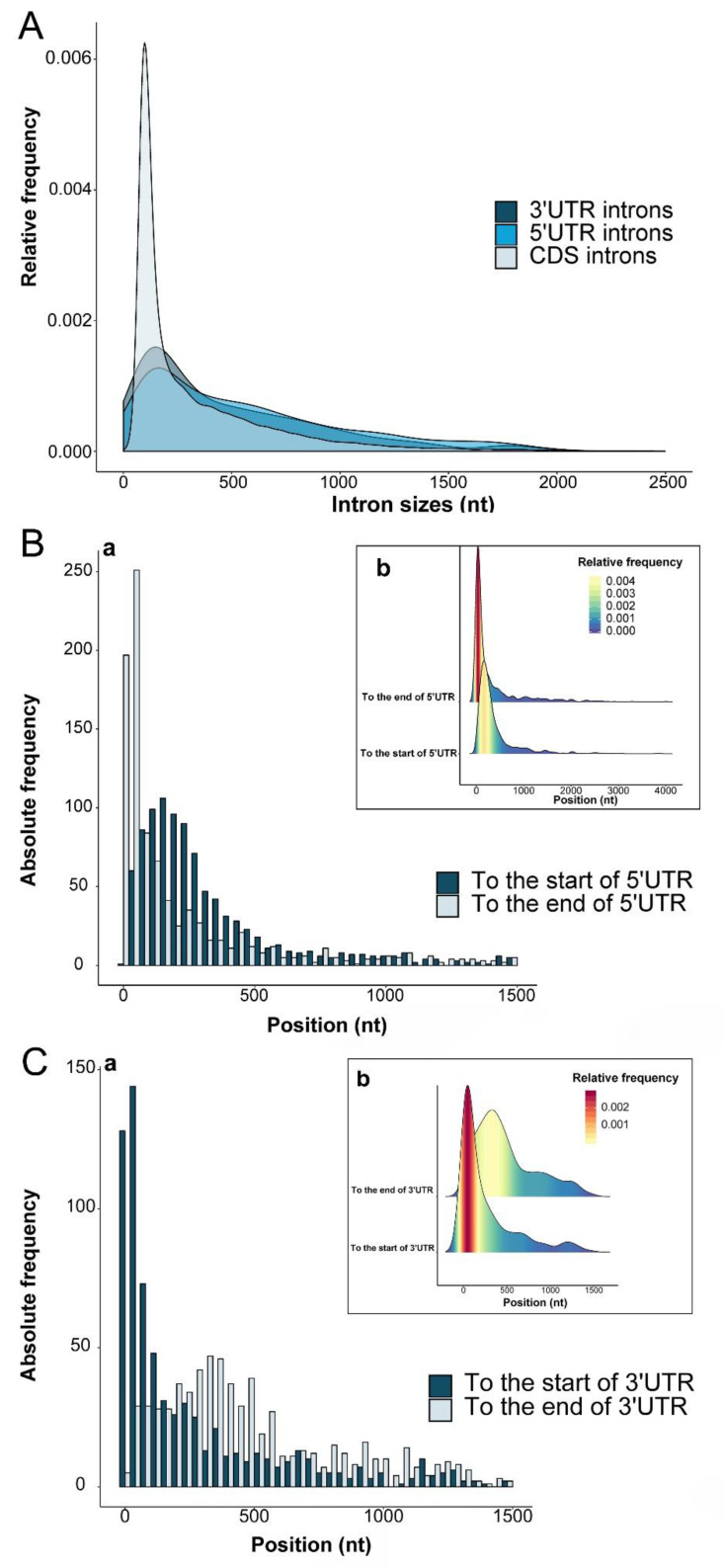
Length distributions of 5′UTR, 3′UTR and CDS introns (**A**) and position distribution of 5′UTR introns (**B**) and 3′UTR introns (**C**) relative to the beginning and end of the associated UTRs. For Figure 2A, the horizontal axis represents the size of the intron, and the vertical axis represents the proportion of the introns of different sizes. For Figure 2B,C, (a) Blue bars represent the observed positions of 5′UTR introns relative to the beginning of the 5′UTR and 3′UTR introns relative to the beginning of the 3′UTR (terminate the codon proximal end). Light blue bars represent the observed positions of 5UIs relative to the end of the 5′UTR (i.e., the start codon ATG) and 3UIs relative to the end of the 3′UTR. (b) Sierra of UIs relative to the end of the UTR and the end of the UTR with color diversity.

Intron number and size distribution analysis result showed that the number and length of introns within 5′UTRs, CDSs and 3′UTRs varied (Figure 2A). The 5UI and 3UI have very similar average length distributions (5′UTR: *n* = 965, mean = 587.5 nucleotides, median = 450 nucleotides, LQ = 168 nucleotides, UQ = 836 nucleotides, SD = 548.3 nucleotides; 3′UTR: *n* = 745, mean = 563.5 nucleotides, median = 335 nucleotides, LQ = 139 nucleotides, UQ = 730 nucleotides, SD = 979.6 nucleotides). Their average length were both higher than the CDS introns (*n* = 148,831, mean = 343.2 nucleotides, median = 171 nucleotides, LQ = 102 nucleotides, UQ = 441 nucleotides, SD = 454.9 nucleotides).

By comparing the intron size distributions within 5′UTRs, 3′UTRs, and CDSs, it is obvious that short introns accounted for a lot, which is similar to the result in Arabidopsis [30]. Most abundant short introns <50 nucleotides were found in the 3′UTRs, followed by in the 5′UTRs, and in CDSs short introns were very rare. The relative frequency of introns with length ranging from 100 to 300 nucleotides in CDSs was significantly higher than that in 5UIs and 3UIs. Longer introns above 300 nucleotides in UIs were more than that in CDSs, indicating that CDS intron length was more conservative.

Figure 2B,C respectively represents the distribution of intron position within the 5′ UTRs and 3′UTRs, relative to the beginning and end of the corresponding UTRs. It appears that 5UIs are preferentially located close to the stop ends of 5′UTRs (nearby the translation initiation site of genes), although the location preference close to the beginning of 5′UTR is also relatively high. The splicing-dependent complex mRNA-protein (mRNP) component, which showed positive influence on rapid export and translation of newly synthesized mRNA, is also deposited as close as possible to the 5′ end of the mRNA [35]. Studies have also shown that 5UI would lead to large accumulation of EJCs, which interact with the translation initiation site and result in IME [36,37]. The proximity of 5UI to the end of 5′UTR (which is also close to the translation start site and 5′ end of mRNA) is well consistent with the 5UI regulatory role in gene translation [30].

It was reported that an EJC downstream of the stop codon should persist and stimulate NMD [38]. In our present study, we found that 3UIs are more frequently located at the beginning of 3′UTRs, i.e., close to the stop codon. The proximity of 3UI to the stop codon might cause the stop codon from being recognized as premature and triggering NMD [39].

**Figure 3:**
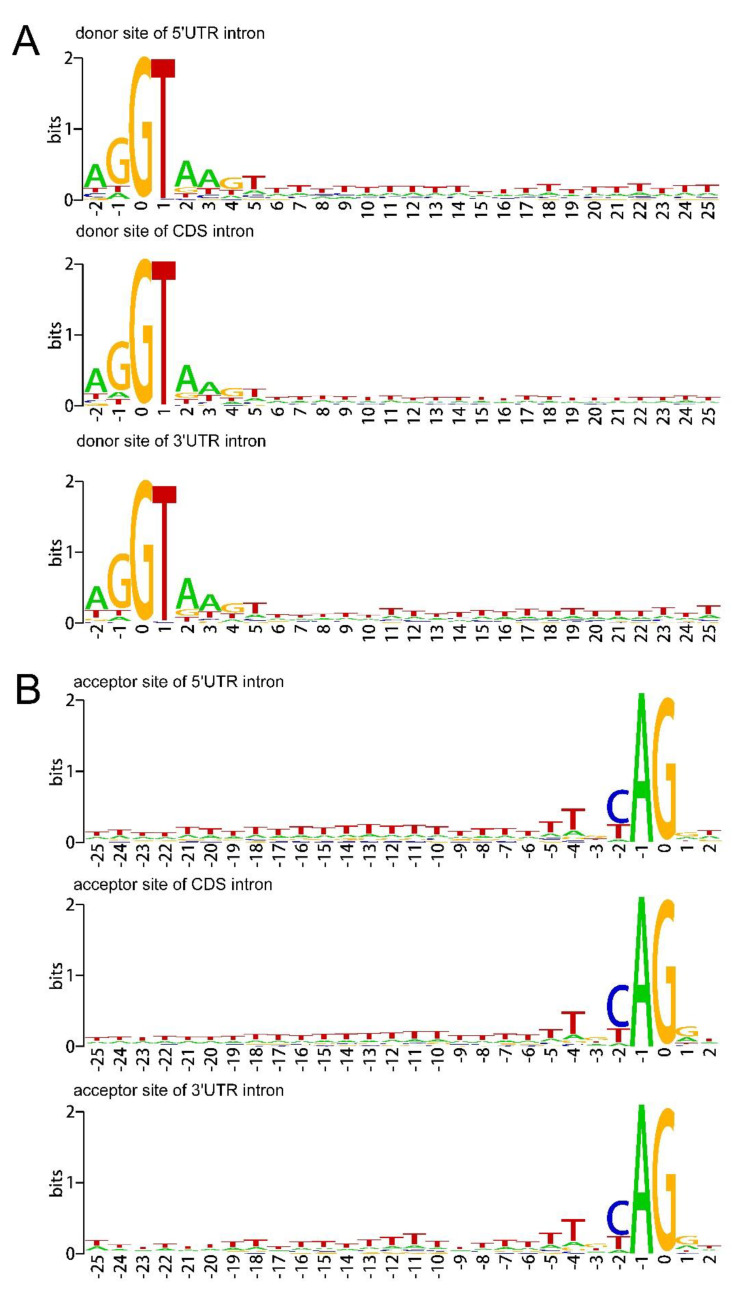
Sequence logos showing the nucleotide bias around the donor site (**A**) and acceptor site (**B**) of 5′UTR, CDS, and 3′UTR introns. The sequence marker shows the nucleotide deviation around the donor site and acceptor site of the 5′UTR, CDS, and 3′UTR introns. The x-axis refers to the base at the beginning of the intron, and the letter height reflects the nucleotide deviation at each position. Only 5 nucleotide exons and 25 nucleotide intron sequences of the donor site and only 2 nucleotide exons and 25 nucleotide intron sequences of the acceptor site are included in the sequence identifier, because the nucleotide usage outside of these regions is not significantly different from the background level.

Intron removal is influenced by many splicing signals and factors [40]. The splice sites (SS) in the exon-exon junction, including 5′ donor site and 3′ acceptor site, were usually conserved. However, in some instances the SS alternate, resulting in AS event [41]. By comparing the nucleotide preferences surrounding the splicing junction using sequence logos [42], the nucleotide bias around the donor and acceptor site of 5′UTR, CDS and 3′UTR introns were found. Figure 3A,B respectively shows the aggregation of nucleotides around the splice donor (GT) and splice acceptor (AG) junctions in 5′UTRs and 3′UTRs. About 97.92% of the 5UIs were with splice site pair of GT-AG, followed by GC-AG (1.45%), AT-AC (0.20%), CT-AC (0.20%), CT-GG (0.10%), and TG-AT (0.10%). Of the 3UIs, 97.18% were with splice site pair of GT-AG, followed by GC-AG (2.14%), AT-AC (0.26%), GT-TG (0.13%), TA-AG (0.13%), and TT-AG (0.13%). The splice site pair category results of sweet orange UIs were similar to the findings in Arabidopsis and mammalian genomes [30,43]. The sequence logos showed that both the UTR introns and the CDS introns exhibit A/T-rich element in both donor sites and receptor sites. The early recognition of introns is thought to be mediated by UA-binding proteins [40]. Thus, it was suspected that the A/T-rich element might contribute to intron recognition. It was reported that U-rich sequence in the 5UI can bind to the RNA-binding protein, which can promote the translation initiation of uAUG by interacting with transcription initiation factors [44,45,46,47]. This suggests that the A/T-rich element in the 5UI contribute to gene translation initiation. Additionally, Chung et al. [30] identified a significant C-rich region near the donor site of Arabidopsis 5UI, which was predicted to be necessary for the spliceosomal recognition of introns within non-coding sequences. Although a C-rich region (ranges from +5 to +25 bases after intron start) can also be found in sweet orange 5UIs, the frequency of C is much lower.

### 2.2. Functional Implications of Genes with UTR Introns

To explore the functional characteristics of 5UI-Ts and 3UI-Ts, GO enrichment analysis was performed. From the aspect of biological processes, 107 and 7 GO terms were significantly enriched for 5UI-Ts and 3UI-Ts, respectively (Supplemental Materials Tables S2 and S3). For the 5UI-Ts, genes involved in “translation”, “peptide biosynthetic process”, “metabolic process”, and “gene expression” accounted a lot. While, 3UI-Ts were mainly involved in “defense response”, “response to stress”, “response to stimulus”, “histone methylation”, “peptidyl-lysine methylation”, “histone lysine methylation”, and “peptidyl-lysine modification”. From the aspect of cellular component, 41 GO terms, including genes involved in “organelle”, “ribosome”, “intracellular ribonucleoprotein complex”, “intracellular part”, “cell part”, and “cell” were significantly enriched for 5UI-Ts. For the 3UI-Ts, there are just 4 terms, i.e., “exocyst”, “cell cortex”, “cell cortex part”, and “cytoplasmic region”, were identified to be significantly enriched. From the aspect of molecular function, 45 and 12 GO terms were significantly enriched respectively for 5UI-Ts and 3UI-Ts. For the 5UI-Ts, genes were mainly involved in “protein binding”, “zinc ion binding”, “methionine adenosyltransferase activity”, “glycylpeptide N-tetradecanoyltransferase activity”, “ribonuclease inhibitor activity” “myristoyltransferase activity”, “structural constituent of ribosome”, “translation factor activity”, “RNA binding”, and so on. For the 3UI-Ts, genes were found to involved in “ADP binding”, “protein binding”, “binding”, “histone binding” and “translation factor activity, RNA binding”, and so on. The GO enrichment analysis results indicated that the 5UI-Ts were highly correlated with the gene expression, while many 3UI-Ts were mainly involved in stress responses. Moreover, the main functions of 5UI-Ts and 3UI-Ts differed and their roles in different cell parts varied.

By using KEGG enrichment analysis, we investigated the pathway enrichment of both 5UI-Ts and 3UI-Ts (Supplemental Materials Table S4 and S5). It has been confirmed that the presence or absence of a 5UIs can determine the mRNA export mechanism [23,48]. Consistently, the only significantly enriched pathway for 5UI-Ts is “RNA transport”. Genes such as *translation initiation factor protein EIF1* (Cs4g10310.1, Cs4g10310.2), *EIF1A* (Cs4g10310.1, Cs4g10310.2) and *EIF5* (Cs3g18950.1, Cs6g18000.1, Cs6g18000.2), *protein transport proteins* (*SEC13*, Cs1g15600.1 and Cs2g28780.1), *nonsense-mediated mRNA decay protein 3* (*NMD3*, Cs6g17980.1), and *Ran GTPase-activating protein 1* (*RANGAP1*) (Cs9g06440.1, Cs9g06440.2, and Cs9g06440.3) which were identified to be RNA transport related. Notably, EIF1 and EIF5 have been revealed to function in stimulating the assembly of the translation initiation complex by interacting with the 40S ribosomal subunit [49]. SEC13, as a component of the nuclear pore complex (NPC) and the COPII coat, has been proved to be required for efficient mRNA export from the nucleus to the cytoplasm and for correct nuclear pore biogenesis and distribution [50]. *NMD3*, however, was found to be involved in NMD of mRNAs containing premature stop codons [51]. The RANGAP1 converts cytoplasmic GTP-bound RAN to GDP-bound RAN, which is essential for RAN-mediated nuclear import and export [52]. From these reports, it can be concluded that these RNA transport related genes were all involved in gene expression regulation. The existence of UI in these genes indicated that UI play roles in regulating gene expression.

For 3UI-Ts, only “splicesome” pathway showed significant enrichment through KEGG pathway enrichment analysis. The significant enrichment of splicesome related gene was consistent with the fact that 3UI-Ts would be rapidly degraded through NMD [23,24,25]. The splicesome related 3UI-Ts included one *small nuclear ribonucleoprotein B and B’* gene transcript (*SNRPB*, Cs2g10375.1), four *heterogeneous nuclear ribonucleoprotein A1/A3* gene transcripts (*HNRNPA1*, Cs6g16060.1, Cs7g25330.1, Cs7g25330.2 and Cs7g25330.3), one *ATP-dependent RNA helicase DDX46/PRP5* gene transcript (*DDX46*/*PRP5*, orange1.1t03258.1), two *U4/U6.U5 tri-snRNP-associated protein 3* gene transcripts (*SNRNP27*, Cs4g03590.1, and Cs4g03590.2), and two *pre-mRNA-splicing factor* gene transcripts (*ISY1*, Cs2g25120.1 and Cs2g25120.2). Their roles in pre-mRNA splicing have been well demonstrated [52,53,54,55,56,57], which is to support the 3UI function in regulating gene expression.

We further applied PageMan to show the pathway enrichment using the corresponding genes of the 5UI-Ts, 3UI-Ts and UI-Ts with both 5UI and 3UI (respectively 617, 469 and 74 genes). PageMan enrichment analysis showed that for the 5UI and 3 UI containing genes, most genes were categorized into “not assigned” pathway (Supplemental Materials Tables S6 and S7). 

For the 5UI containing genes, more than 30 genes were found to be involved in “protein” (92/617, 14.91%), “RNA” (86/617, 13.94%), “RNA. regulation of transcription” (76/617, 12.32%), “protein. degradation” (50/617, 8.10%), “protein. degradation. ubiquitin” (46/617, 7.46%), “protein. degradation. ubiquitin. E3′ (38/617, 6.16%) and “signalling” (30/617, 4.86%) (Table 4). Studies have indicated that 5UI of *AtMHX* may have a special contribution to translation efficiency [58]. Promoter with 5UI can improve higher gene expression and product accumulation compared with promoter without it [14,15,16]. From the PageMan pathway enrichment result of 5UI containing genes, it is easy to find that these genes are mainly involved in gene expression, including both gene transcription and gene translation, suggesting that 5UI function at both transcriptional and post-transcriptional levels.

For the 3UI containing genes, however, pathways involving more than 30 genes include “not assigned. no ontology. pentatricopeptide (PPR) repeat-containing protein” (61/469, 13.01%), “stress” (44/469, 9.38%), “stress. biotic” (41/469, 8.74%), “RNA” (39/469, 8.32%), “stress. biotic. PR-proteins” (37/469, 7.89%), “protein” (31/469, 6.61%) and “RNA. regulation of transcription” (31/469, 6.61%). Notably, among these 3UI-containing genes, 61 (13.01%) were *pentatricopeptide repeat-containing proteins* (*PPRPs*) genes (Table 4). Proteins containing PPR motifs play a role in transcription, RNA processing, splicing, stability, editing, and translation [59]. PPRP is thought to be the main mediator of post-transcriptional regulation of organelles [60]. Most PPRPs are localized in mitochondria or chloroplasts. Dahan and Mireau [61] reported that mitochondrial PPRPs act by preventing translation or accumulation of mitochondrial transcripts, and their gene products can induce cytoplasmic male sterility mutants to embryonic developmental defects and cytoplasmic male sterility. In addition, PPRPs have been identified to play a role in translational and post-translational processes in response to biotic and abiotic stresses [62], suggesting that 3UIs play important roles in regulating the expression of stress responsive gene. Consistently, 41 (8.74%) out of the 3UI containing genes, were *disease resistance* (*R*) genes. And some *R* genes (such as Cs1g15550.1, Cs1g17000.1 and Cs1g16990.1) were found to contain multiple introns. Plant *R* genes are involved in pathogen recognition and subsequent activation of innate immune responses [63]. The abundance of disease resistance genes again supported the regulatory role of 3UIs in the expression of stress responsive genes.

The genes containing both 5UI and 3UI were mainly involved in “RNA” (12/74, 16.22%), “RNA. regulation of transcription” (10/74, 13.51%), “protein” (9/74, 12.16%), “not assigned. no ontology. Pentatricopeptide repeat (*PPR*)-containing protein” (7/74, 9.46%) and “signaling” (5/74, 6.76%) (Supplemental Materials Table S8).

### 2.3. UTR Introns and Transcriptional Enhancers

Transcriptional enhancers have been identified in intron sequences by computational methods [64,65,66]. Cenik et al. [28] found genes with regulatory roles are particularly enriched with 5UIs compared to genes without 5UI. Therefore, UIs can be considered as important *cis*-regulatory elements that regulate the multiple levels of gene expression. In this study, we predicted the *cis*-acting elements in UI sequences. Totally, 39893 and 28702 *cis*-acting elements are respectively identified in 5UIs and 3UIs. Among these *cis*-acting elements, “core promoter elements around -30 of transcription start” and “common *cis*-acting element in promoter and enhancer regions” take the largest part, and more than 88% 5UIs and 3UIs contain these elements.

It has been found that introns present in the 5′UTR often lead to increased expression of transgenes [64,65,66,67]. Grant et al. [16] found that the synthetic 5′ UTR intron fragments of the *Glycine max polyubiquitin* (*Gmubi*) gene placed downstream of the 35S core promoter could enhance the expression of transgene, suggesting that these fragments can function as promoter regulatory elements and contribute to increased expression. The existence of promoter elements in UIs suggested that UIs function similarly to promoters in regulating gene expression. Besides, “light responsive element”, “*cis*-acting regulatory elements essential for the anaerobic induction”, and many phytohormone responsive elements were also widely found in UI sequences (Supplemental data Tables S9 and S10), suggesting that UIs might function in the light or phytohormone responses.

### 2.4. Experimental Validation of 5UI and 3UI Splicing

**Figure 4:**
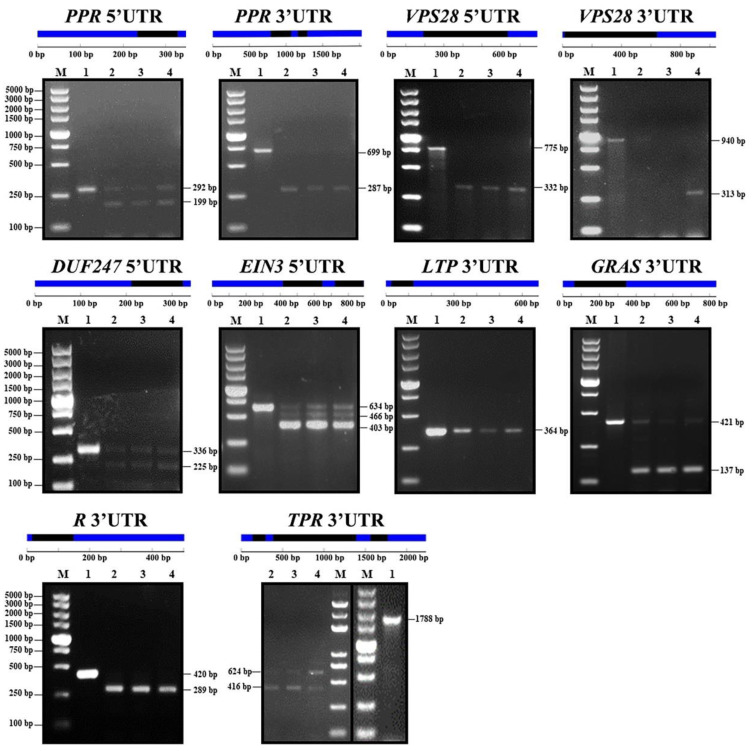
Electrophoresis detection results of the UTR introns (UIs) in untranslated regions (UTRs) of the eight selected genes. 1~4 respectively represents the PCR products using leaf gemomic DNA (gDNA), root complementary DNA (cDNA), leaf cDNA, and stem cDNA as template, respectively. M: DL5000 Marker; The UTR structure is available on the GSDS online website (http://gsds.cbi.pku.edu.cn/), with blue for exons and black for introns in UTR. *PPR*: *Pentatricopeptide repeat superfamily protein* (Cs6g01290.1), *VPS28*: *Vacuolar protein sorting-associated 28* (Cs2g06750.1), *EIN3*: *Ethylene-insensitive 3* (Cs2g29100.1), *DUF247*: *domain of unknown function 247* gene (Cs2g24990.1), *GRAS*: *GRAS transcription factors* (Cs8g18700.1), *TPR*: *Tetratricopeptide repeat-like superfamily protein* (Cs8g15200.1), *R*: *Disease resistance protein* (Cs5g21990.1) and *LTP*: *Lipid transfer protein* (Cs5g09070.2).

By using leaf gDNA and cDNAs of root, leaf and stem as templates, PCR reactions were performed to verify the UI existence in UTRs. The results proved the existence of introns in the UTRs and intron retention events were found in some UI-Ts (Figure 4). 

Two 3UIs, one 5UI and one 3UIs were annotated respectively in *PPR* (Cs6g01290.1), *VPS28* (Cs2g06750.1) 5′UTR, *R* gene (Cs5g21990.1) 3′UTR, our RT-PCR result showed that the cDNA sequence did not contain these introns in all the three organs, which meant they were all removed during the forming of mature mRNA. While, for the one 3UI containing *LTP* gene (Cs5g09070.2), intron retention was found in all the organs. Moreover, there was one 5UI, one 5UI and one 3UI annotated in the *PPR* gene (Cs6g01290.1) 5′UTR, *DUF247* gene (Cs2g24990.1) 5′UTR, and *GRAS* gene (Cs8g18700.1) 3′UTR respectively. However, by using RT-PCR, two bands were amplified from cDNA. Among the two bands, one was the same as the gDNA sequence and the other one was shorter than the gDNA sequence. The removed sequence length was the same as the length of the UI. This indicated that, for these three UTRs, intron retention happened in some gene transcripts.

The *VPS28* gene also contained one 3UI. According to the RT-PCR result, we found that 3′UTR amplified from root cDNA is the same length as the gDNA sequence. The 3′UTR amplified from stem cDNA, however, was shorter than the gDNA, and the missed length was same to the length of the 3UI. The result indicated that intron transcription of the *VPS28* 3′UTR differed in these two organs. Moreover, no band was amplified from the leaf cDNA, suggesting that the expression of *VPS28* 3′UTR in leaf was so low that it could not be successfully amplified by RT-PCR.

Two 5UIs, one was 231 bp and the other 168 bp, were annotated in the *EIN3* gene (Cs2g29100.1). By using RT-PCR, we amplified tree bands from the cDNAs of the three organs. The length of largest band was same as the gDNA, the lacked length of the middle one compared with gDNA was the length of the small intron, while the missing length of smallest one was the length of the big intron. 

There are three 3UIs annotated in the *TPR* gene (Cs8g15200.1), and their length was 1006 bp, 208 bp and 158 bp, respectively. By using RT-PCR, we amplified two target bands, one was 1164 bp (the length of the largest 3UI plus the smallest 3UI) shorter than gDNA and the other 1372 bp (the total length of all the three 3UIs) shorter than gDNA.

**Figure 5:**
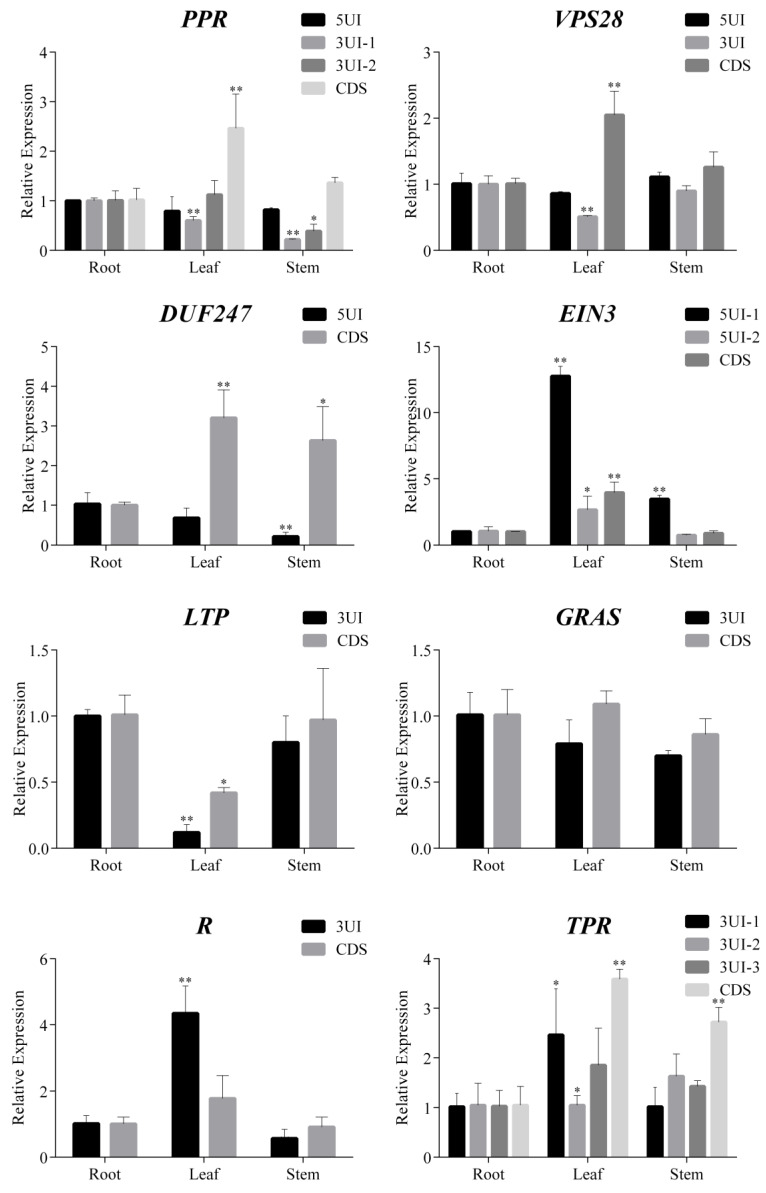
Relative expression results of genes and their UTR introns (UIs) in *Citrus sinensis* root, leaf and stem. * and ** respectively represent significant difference (*p* < 0.05) and very significant difference (*p* < 0.01) compared with root. 5UI: intron in the 5′UTR; 3UI: intron in the 3′UTR; CDS: coding sequence; represent the relative expression levels of genes containing these structures. *PPR*: *Pentatricopeptide repeat superfamily protein* (Cs6g01290.1), *VPS28*: *Vacuolar protein sorting-associated 28* (Cs2g06750.1), *EIN3*: *Ethylene-insensitive 3* (Cs2g29100.1), *DUF247*: *domain of unknown function 247 gene* (Cs2g24990.1), *GRAS*: *GRAS transcription factors* (Cs8g18700.1), *TPR: Tetratricopeptide repeat-like superfamily protein* (Cs8g15200.1), *R*: *Disease resistance protein* (Cs5g21990.1) and *LTP*: *Lipid transfer protein* (Cs5g09070.2).

To further study the expression of UIs in different organs and to investigate the correlation between the expression of UIs and their corresponding genes, qRT-PCR analysis of the 8 selected genes and all the UIs annotated in them in three sweet orange organs, i.e., root, stem and leaf, was performed (Figure 5). The qRT-PCR results revealed expression of all the UIs in all the three organs, which is different from the RT-PCR results. This might be caused by the sensitivity difference between the two techniques.

*PPR* gene contained one 5UI and two 3UIs, the gene’s expression in leaf was significantly higher than in the root, the expression of 3UI-1 in leaf and stem was very significantly lower than in the root, the expression of 3UI-2 in stem was significantly lower than in the root. *VPS28* gene contained one 5UI and 3UI, the gene’s expression in leaf was significantly higher than in the root, while the expression of 3UI in leaf is significantly lower than in the root. *DUF247* gene contains one 5UI, the gene’s expression in leaf and stem was respectively very significantly and significantly higher than in the root, while the 5UI expression in stem is very significantly lower than in the root. *EIN3* gene contains two 5UIs, the gene’s expression in leaf is very significantly higher than in the root. The expression of 5UI-1in both leaf and stem were significantly higher than in the root. The expression of 5UI-2 in leaf was significantly higher than in the root. *LTP* gene contains one 3UI, the gene’s expression in leaf was significantly lower than in the root, and the 3UI’s expression significantly lower than in the root. *GRAS* gene contains one 3UI, the expression of the gene and 3UI showed no significant difference among the three organs. The *R* gene contains one 3UI, the gene’s expression showed no significant difference among the three organs, but the expression of its 3UI in leaf and stem was significantly higher than in the root. *TPR* gene contains three 3UIs, the gene’s expression in leaf and stem was significantly higher than in the root, and the expression of 3UI-1 in leaves significantly higher than in the root, but other two UIs showed no significant difference in the three organs.

It has been reported that the 5UI expression function in enhancing gene expression, and 3UI expression usually lead to the degradation of its corresponding gene [13,68,69,70,71]. Consistently, in our present study, we found that the expression of *VPS28* and its 3UI was significantly negative correlated. Although no significant correlation was identified, the relative coefficient between the expression of *EIN3* and its two 5UIs was positive. However, we also found that the expression of *R* gene and its 3 UI were significantly positive correlated. The relative coefficient between the expression of *LTP* and its 3UI, *TPR* and its three 3UIs was positive, the expression of *PPR* and its 5UI, *VPS28* and its 5UI, *DUF247* and its 5UI, was negative. This might be due to the very complex nature of gene expression regulation and its involvement of many sequence elements, noncoding RNAs or factors [72,73,74]. Thus, the detailed regulatory role of UIs in gene expression needs to be further studied by experimental researches.

## 3. Materials and Methods

### 3.1. Genome-Wide Identification of Sweet Orange UIs

Genome data of *C. sinensis* was downloaded from the *Citrus sinensis* annotation project (http://citrus.hzau.edu.cn/orange/download/index.php/) [31,75]. Based on the genome annotation data, introns in CDSs, 5′UTR and 3′ UTR were extracted. As many genes having alternative splicing events, intron retention in any transcript was excluded in order to identify all the UTR introns. Statistical analysis of UI density, position preference, length and nucleotide composition was performed using Perl. And the ggplot2 software was used for the figures drawing.

### 3.2. Enrichment Analysis of Genes Containing UIs

To illustrate the genes containing UIs, we conducted GO and KEGG pathway enrichment analyses of the 5UI containing and 3UI containing genes on the Dynamic GO Enrichment Analysis online website (https://www.omicshare.com/tools/Home/Report/goenrich) and on the dynamic KEGG enrichment analysis online website (https://www.omicshare.com/tools/Home/report/koenrich), respectively. Moreover, to better show the pathway enrichment result, MapMan and its embedded PageMan software were used [76]. 

### 3.3. Cis-Acting Element Prediction of UI Sequences

Intron regulatory elements play important roles in regulating gene expression [16]. In this study, the *cis*-acting elements in UIs were analyzed and retrieved using PlantCARE (http://bioinformatics.psb.ugent.be/webtools/plantcare/html/) to show the distribution of *cis*-acting elements in UI sequences.

### 3.4. UI Structure Verification

To verify the UI structure (existence and expression pattern) in the identified UI containing genes, eight representative genes, including a *Pentatricopeptide repeat superfamily protein* (*PPR*, Cs6g01290.1), a *Vacuolar protein sorting-associated 28* gene (*VPS28*, Cs2g06750.1), an *ethylene-insensitive3* (*EIN3*, Cs2g29100.1), a *domain of unknown function 247* gene (*DUF247*, Cs2g24990.1), a *GRAS transcription factor* (*GRAS*, Cs8g18700.1), a *Tetratricopeptide repeat-like superfamily protein* (*TPR*, Cs8g15200.1), a *Disease resistance protein* (*R*, Cs5g21990.1), and a *Lipid transfer protein* (*LTP*, Cs5g09070.2), were selected and subjected to RT-PCR and qRT-PCR analysis. Among these genes, *EIN3* and *DUF247* was predicted to contain 5UI, *GRAS*, *TPR*, *R,* and *LTP* only contain 3UI, and *PPR* and *VPS28* contain both 5UI and 3UI.

Leaf, stem and root samples of Four-month-old Valencia sweet orange (*Citrus sinensis* cv. Valencia) were collected, pre-cooled in liquid nitrogen and then stored at −80 °C for further gDNA and RNA extraction. For DNA isolation, E.Z.N.A.^®^ HP Plant DNA kit (Omega, Norcross, GA, USA) was used. For total RNA isolation, the total RNA extraction kit (TIANGEN, Beijing, China) was used. After RNA integrity and quality check using 1% agarose gel electrophoresis and spectrophotometry, high quality RNA samples were reverse-transcribed into cDNA using the First Strand cDNA Synthesis Kit (Thermo Scientific, Wilmington, DE, USA). The results of the validation showed that none of the UIs of some genes were successfully cloned. The extracted gDNA and reverse-transcribed cDNA of different tissues were used as templates to amplify by RT-PCR verification. Primers used for UI structure verification were designed according to the UTR sequences of each UI containing gene transcripts to amplify sequence containing all the possible UIs. Primer information was listed in Supplemental Materials Table S1.

To study the expression level of UI in different organs of sweet orange, qRT-PCR was performed to show the expression pattern of UIs of the selected eight genes using the same RNA for UI verification. cDNA for qRT-PCR was obtained using the RNA samples using Hifair^®^ Ⅱ 1st Strand cDNA Synthesis SuperMix kit. Reactions were carried out on the LightCycler480 Real-time PCR in a final volume of 20 µL containing 1 µL of cDNA, 10 µL Hieff^®^ qPCR SYBR^®^ Green Master Mix (No Rox/ Low Rox/ High Rox), 0.8 µL each of the forward and the reverse primers (2 µM), and 7.4 µL of sterile water. The thermocycler was programmed as: 95 °C for 5 min followed by 40 cycles of 95 °C for 10 s, 54~58 °C for 20 s and 72 °C for 20 s. The expression was calculated by 2^−∆∆Ct^ method [77] with *β-actin* gene as internal control [78]. Data processing and differential significance analysis were performed using Excel2016 and SPSS17.Information of primers used for qRT-PCR analysis was also shown in Table S11.

## 4. Conclusions

In this study, we identified a total of 965 5UI sequences and 745 3UI sequences from 825 5UI-Ts (corresponding to 617 genes) and 570 3UI-Ts (469 genes). Among these UI-Ts, 77 (74 genes) contain both 5UI and 3UI. The density of 5UI and 3UI was respectively the highest in chromosome 2 and chromosome 1, and were both the lowest in chromosome On chromosome 2 and chromosome 6, the density of 5UI was found to be significantly greater than 3UI. The average length of 5UIs and 3UIs were similar and were both larger than CDS introns. 5UIs were more biased towards the both ends especially close to the stop end of the 5′UTR, and 3UIs were biased towards the start of 3′UTR. Both the UTR introns and the CDS introns exhibit A/T-rich element, which is considered to be an efficient splicing intron [40,79]. Function enrichment analysis revealed that genes containing 5UI were significantly enriched in the RNA transport pathway, which supported their role in mRNA export [23]. Genes containing 3UI were significantly enriched in splicesome, suggesting that the 3UIs to be widely involved in the splicing of pre-mRNA or NMD [69,80,81]. Notably, many *PPRPs* and *R* genes were identified to be UI-containing, indicating that UIs contribute to the expression regulation of these two gene families and play roles in the plant responses to biotic and abiotic stresses during translation and post-translational processes [62,82,83]. The regulatory role of UIs in the expression of genes containing UIs, especially the *R* genes, can be further studied. Moreover, many promoter enhancing related elements were identified in the UIs. The existence of these sequences demonstrated their regulatory roles in gene expression. Additionally, the expression of UIs in gene transcripts was confirmed by using RT-PCR and qRT-PCR. To our knowledge, this is the first report about the identification and characterization of horticultural plants from genome-wide level. The results obtained from this study could provide evidences for further understanding of the role of UIs in regulating gene expression.

## Figures and Tables

**Table 1:** Statistics information of 5′ untranslated regions (UTR), coding sequences (CDSs) and 3′ UTR in *Citrus sinensis*.

	Number of Sequences	Sequences with Introns	Total Bases (Genomic)	Intron/Sequence	Number of Introns/Nucleotide (mRNA)
5′UTR	16,916	617	6.8 × 10^6^	0.06	1.42 × 10^−4^
CDS	23,394	17,897	3.8 × 10^7^	4.81	2.98 × 10^−3^
3′UTR	17,408	469	1.2 ×10^7^	0.04	6.36 × 10^−5^

**Table 2:** Information of the UTR-intron (UI) numbers in the 5UI and 3UI containing gene transcripts (respectively abbreviated as 5UI-Ts and 3UI-Ts). 1 UI~6 UIs respectively means that there is 1~6 UIs present in the UTR. There are a total of 825 gene transcripts containing 5UI, and a total of 570 gene transcripts containing 3UI. NS: not shown.

UI Number	5UI-Ts Number/Percentage (Gene ID)	3UI-Ts Number/Percentage (Gene ID)
1 UI	713/86.43% (NS)	448/78.60% (NS)
2 UIs	90/10.91% (NS)	90/15.79% (NS)
3 UIs	18/2.18% (Cs1g06160.1, Cs2g16080.1, Cs2g03700.1, Cs2g01050.1, Cs3g12240.1, Cs3g19040.1, Cs4g10860.1, Cs5g07860.1, Cs6g06255.1, Cs7g12410.1, Cs9g09475.1, orange1.1t05830.1, orange1.1t01413.1, orange1.1t02679.1, orange1.1t02923.1, orange1.1t04234.1, orange1.1t05909.1, orange1.1t06043.1)	18/3.16% (Cs1g09404.1, Cs1g10030.1, Cs3g02530.1, Cs3g21100.1, Cs3g25390.1, Cs4g03945.1, Cs6g08820.1, Cs7g04620.1, Cs7g09600.1, Cs8g11445.1, Cs8g11845.1, Cs8g15200.1, orange1.1t02481.1, orange1.1t05875.1, orange1.1t03487.1, orange1.1t06018.1, orange1.1t05916.1, orange1.1t05924.1)
4 UIs	2/0.24% (Cs2g17870.1, Cs5g25765.1)	8/1.40% (Cs2g08495.1, Cs3g10260.1, Cs3g12715.1, Cs5g26550.1; Cs5g28645.2, Cs7g15390.1, orange1.1t05956.1, orange1.1t03536.1)
5 UIs	2/0.24% (orange1.1t06039.1, Cs7g06380.1)	4/0.70% (Cs2g11780.1, orange1.1t01460.1, orange1.1t03486.1, Cs1g16990.1)
6 UIs	0	2/0.35% (Cs1g15550.1, Cs1g17000.1)

**Table 3:** Statistics information of the distribution of UTR introns (UIs) and transcripts containing UI (UI-Ts) in the *Citrus sinensis* chromosomes. 5UI-T represents gene transcript containing 5UI; 3UI-T represents gene transcript containing 3UI. Chr: chromosome.

Chr No.	5UI Numbers/Percentage	5UI Density	3UI Numbers/Percentage	3UI Density	5UI-T Number/Percentage	5UI-T Density	3UI-T Number/Percentage	3UI-T Density
chr1	81 (8.39%)	2.81 × 10^−6^	103 (13.83%)	3.58 × 10^−6^	70 (8.49%)	2.43 × 10^−6^	72 (12.6%)	2.5 × 10^−6^
chr2	162 (16.79%)	5.26 × 10^−6^	65 (8.73%)	2.11 × 10^−6^	137 (16.61%)	4.45 × 10^−6^	50 (8.77%)	1.62 × 10^−6^
chr3	87 (9.02%)	3.03 × 10^−6^	83 (11.14%)	2.89 × 10^−6^	73 (8.85%)	2.54 × 10^−6^	61 (10.7%)	2.13 × 10^−6^
chr4	75 (7.77%)	3.75 × 10^−6^	60 (8.05%)	3.00 × 10^−6^	66 (8.00%)	3.3 × 10^−6^	48 (8.42%)	2.40 × 10^−6^
chr5	100 (10.36%)	2.76 × 10^−6^	85 (11.41%)	2.35 × 10^−6^	84 (10.18%)	2.32 × 10^−6^	71 (12.46%)	1.96 × 10^−6^
chr6	96 (9.95%)	4.53 × 10^−6^	39 (5.24%)	1.84 × 10^−6^	85 (10.30%)	4.01 × 10^−6^	33 (5.79%)	1.56 × 10^−6^
chr7	97 (10.05%)	3.01 × 10^−6^	78 (10.47%)	2.42 × 10^−6^	85 (10.30%)	2.64 × 10^−6^	62 (10.88%)	1.93 × 10^−6^
chr8	54 (5.59%)	2.38 × 10^−6^	57 (7.65%)	2.51 × 10^−6^	48 (5.82%)	2.11 × 10^−6^	43 (7.54%)	1.89 × 10^−6^
chr9	48 (4.97%)	2.59 × 10^−6^	27 (3.62%)	1.46 × 10^−6^	43 (5.21%)	2.32 × 10^−6^	23 (4.04%)	1.24 × 10^−6^
chrUn	165 (17.01%)	-	148 (19.86%)	-	134 (16.24%)	-	107 (18.77%)	-

**Table 4:** 5′UTR intron (5UI) and 3′UTR intron (3UI) numbers and length in UTRs of UI-containing *pentatricopeptide repeat containing proteins* (*PPRPs*) and *disease resistance* (*R*) genes.

Gene Family	Gene ID	3UI Number and Length (bp)	5UI Number and Length (bp)
*PPR*	Cs4g02090.2	1 (150)	-
	Cs4g03660.1	1 (119)	-
	Cs4g07420.1	1 (1165)	-
	Cs4g13530.1	2 (366, 98)	-
	Cs4g13560.1	2 (710, 98)	-
	Cs4g20340.4	1 (334)	-
	Cs4g20340.1	-	1 (576)
	Cs4g20340.2	-	1 (143)
	Cs2g05520.1	1 (754)	-
	Cs2g07840.2	1 (486)	-
	Cs2g09470.2	1 (1044)	-
	Cs2g11780.1	5 (614, 1389, 178, 567, 78)	-
	Cs2g13460.1	1 (93)	-
	Cs2g19190.1	1 (93)	-
	Cs2g19710.1	2 (113, 442)	-
	Cs2g27580.1	1 (1233)	-
	Cs5g03910.1	1 (108)	1 (147)
	Cs5g04860.1	1 (422)	-
	Cs5g08440.2	1 (670)	-
	Cs5g17240.1	1 (98)	-
	Cs5g26200.2	1 (771)	-
	Cs5g26550.1	4 (314, 152, 720, 78)	-
	Cs5g34090.1	1 (513)	1 (114)
	Cs7g04230.1	1 (212)	-
	Cs7g04980.1	1 (234)	-
	Cs7g09600.1	3 (781, 330, 112)	-
	Cs7g10230.1	2 (278, 91)	1 (490)
	Cs7g13700.2	1 (949)	-
	Cs7g15390.1	4 (661, 120, 811, 136)	-
	Cs3g02530.2	1 (707)	-
	Cs3g09780.2	1 (103)	-
	Cs3g10260.1	4 (161, 862, 194, 186)	-
	Cs3g11640.1	2 (1094, 97)	-
	Cs3g19210.1	2 (334, 140)	-
	Cs3g20090.1	2 (506, 107)	1 (666)
	Cs3g20090.2	-	1 (462)
	Cs3g20480.1	2 (220, 166)	-
	Cs3g24370.1	2 (104, 668)	-
	Cs3g25390.1	3 (109, 158, 105)	-
	Cs6g01290.1	2 (280, 132)	1 (93)
	Cs6g07760.1	2 (102, 211)	-
	Cs6g08820.2	1 (107)	-
	Cs6g11340.2	1 (270)	-
	Cs6g11530.1	1 (909)	-
	Cs6g11910.2	1 (388)	-
	Cs1g10030.4	1 (194)	-
	Cs1g10310.2	1 (1699)	-
	Cs1g12770.2	1 (997)	-
	Cs1g12780.1	2 (89, 306)	-
	Cs1g24360.1	1 (1265)	-
	Cs1g26320.1	1 (531)	-
	Cs8g15200.1	3 (158, 1006, 208)	-
	Cs8g18540.1	1 (134)	-
	Cs9g01900.1	1 (99)	-
	Cs9g03060.1	1 (468)	-
	Cs9g17260.1	1 (1131)	-
	orange1.1t00940.1	2 (725, 181)	-
	orange1.1t01460.1	5 (431, 636, 473, 134, 97)	1 (268)
	orange1.1t01541.1	2 (554, 509)	-
	orange1.1t04277.2	1 (366)	-
	orange1.1t04409.1	2 (343, 301)	-
	Cs4g03945.1	3 (633, 89, 99)	-
	Cs4g11335.1	1 (295)	-
	Cs9g14456.1	1 (91)	-
*R*	Cs4g07730.1	2 (92, 142)	-
	Cs4g07730.2	2 (87, 138)	-
	Cs4g10830.1	1 (363)	1 (93)
	Cs2g19600.2	1 (238)	-
	Cs2g30590.1	2 (138, 605)	-
	Cs5g20470.1	1 (89)	-
	Cs5g21990.1	1 (131)	-
	Cs5g22710.1	1 (140)	-
	Cs5g28770.1	2 (239, 685)	-
	Cs5g29510.1	1 (145)	-
	Cs7g02220.1	1 (82)	-
	Cs3g13340.1	2 (187, 175)	-
	Cs3g13390.1	2 (180, 130)	-
	Cs1g06720.1	1 (163)	-
	Cs1g08080.2	1 (99)	-
	Cs1g11430.1	2 (291, 1,488)	-
	Cs1g12140.1	1 (482)	-
	Cs1g14030.1	1 (171)	-
	Cs1g14090.1	1 (327)	-
	Cs1g14120.1	1 (400)	-
	Cs1g15550.1	6 (408, 159, 163, 293, 120, 101)	-
	Cs1g16990.1	5 (174, 104, 82, 71, 95 )	-
	Cs1g17000.1	6 (332, 96, 357, 268, 161, 144)	-
	Cs1g18380.2	1 (663)	-
	Cs9g18740.1	1 (178)	-
	orange1.1t01926.1	1 (163)	-
	orange1.1t02481.1	3 (137, 210, 362)	-
	orange1.1t02498.1	1 (290)	-
	orange1.1t02751.1	1 (401)	1 (347)
	orange1.1t02917.1	1 (138)	-
	orange1.1t02924.1	1 (140)	-
	orange1.1t03486.1	5 (169, 195, 93, 377, 127)	-
	orange1.1t03487.3	1 (571)	-
	orange1.1t03742.2	1 (140)	-
	orange1.1t04592.1	1 (238)	-
	Cs2g30865.1	1 (303)	-
	Cs1g09404.1	3 (133, 117, 473)	-
	orange1.1t05891.1	1 (4655)	-
	Cs4g17710.1	1 (712)	-
	Cs4g08050.1	1 (282)	-
	Cs4g08050.2	1 (274)	-
	Cs4g08110.2	1 (277)	-
	Cs6g19070.1	1 (751)	-
	Cs1g14090.2	1 (321)	-
	Cs1g14090.3	1 (321)	-
	orange1.1t03332.1	1 (133)	-

## Data Availability

All the data generated or analyzed during this study are included in this published article and its Supplemental data.

## Supplementary Materials

The following are available online at https://www.mdpi.com/1422-0067/21/9/3088/s1, Table S1: Information of the 5UIs and 3UIs identified in the sweet orange (Citrus sinensis) genome and the gene transcripts containing them. Table S2: Gene Ontology (GO) enrichment analysis result of 5UI-Ts. Table S3: Gene Ontology (GO) enrichment analysis result of 3UI-Ts. Table S4: KEGG pathway enrichment analysis result of 5UI-Ts. Table S5: KEGG pathway enrichment analysis result of 3UI-Ts. Table S6: PageMan pathway enrichment analysis of the 617 genes containing 5UI. Table S7: PageMan pathway enrichment analysis of the 469 genes containing 3UI. Table S8: PageMan pathway enrichment analysis of the 74 genes containing both 5UI and 3UI. Table S9: Information of the identified cis-acting elements in both 5UI sequences. Table S10: Information of the identified Cis-acting elements in both 3UI sequences. Table S11: Information of the 5UIs and 3UIs identified in the sweet orange (Citrus sinensis) genome and the gene transcripts containing them.

## Abbreviations

CDS	Coding sequence
UTR	Untranslated region
UI	UTR intron
RT-PCR	Reverse transcription PCR
qRT-PCR	Quantitative real time PCR
EJC	exon-junction complex
IME	Intron-mediated enhancement
NMD	nonsense-mediated decay
NPC	Nuclear pore complex
UI-Ts	UI-containing gene transcripts
AS	Alternative splicing
SS	Splice sites
